# Supplementary material for: LASSO Regression-Based Diagnosis of Acute ST-Segment Elevation Myocardial Infarction (STEMI) on Electrocardiogram (ECG)
Source: J Clin Med. 2022 Sep 15;11(18):5408. doi: 10.3390/jcm11185408 (PMC9505979; doi:10.3390/jcm11185408)
Supplement: Supplementary file 1 [file jcm-11-05408-s001.zip › jcm-1835037-supplementary.pdf]

Supplementary Tabel S1 The interpretation of ECG features

Abbreviation	Interpretation
CVD family history	cardiovascular disease family history
WBC	white blood cell count
RBC	red blood cell count
HGB	hemoglobin concentration
PLT	platelet count
ALB	albumin
GLB	globulin
K	potassium
Na	sodium
Ca	calcium
GLU	fasting blood glucose
BUN	blood urea nitrogen
CREAT	Serum creatinine
CHOL	total cholesterol
TG	triglyceride
HDL-C	high density lipoprotein cholesterol
LDL-C	low density lipoprotein cholesterol
CK-MB	creatine kinase-MB
Minimum HR	minimum heart rate
Maximun HR	maximum heart rate
Average HR	average heart rate
PR	PR interval
P wave	P wave duration
QRS complex	QRS complex duration
T wave	T wave duration
QT interval	QT interval
QTc interval	QTc interval
QRS axis	QRS axis
RV1+SV5	R wave amplitude of V1 plus S wave amplitude of V5
RV5+SV1	R wave amplitude of V5 plus S wave amplitude of V1
R-PB	Peak R wave to the beginning of P wave
R-P	Peak R wave to peak P wave
R-PE	Peak R wave to the ending of P wave
R-Q	Peak R wave to peak Q wave
R-S	Peak R wave to peak S wave
R-TB	Peak R wave to the beginning of T wave
R-T	Peak R wave to the peak of T wave
R-TE	Peak R wave to the ending of T wave

Q-P	Peak Q wave to peak P wave
S-T	Peak S wave to peak P wave
Q-PB	Peak Q wave to the beginning of P wave
S-TE	Peak S wave to the ending of T wave
I (PB)	the amplitude of the beginning of P wave in Lead I
II(PB)	the amplitude of the beginning of P wave in Lead II
III(PB)	the amplitude of the beginning of P wave in Lead III
aVR(PB)	the amplitude of the beginning of P wave in Lead aVR
aVL(PB)	the amplitude of the beginning of P wave in Lead aVL
aVF(PB)	the amplitude of the beginning of P wave in Lead aVF
V1(PB)	the amplitude of the beginning of P wave in Lead V1
V2(PB)	the amplitude of the beginning of P wave in Lead V2
V3(PB)	the amplitude of the beginning of P wave in Lead V3
V4(PB)	the amplitude of the beginning of P wave in Lead V4
V5(PB)	the amplitude of the beginning of P wave in Lead V5
V6(PB)	the amplitude of the beginning of P wave in Lead V6
I (PE)	the amplitude of the ending of P wave in Lead I
II(PE)	the amplitude of the ending of P wave in Lead II
III(PE)	the amplitude of the ending of P wave in Lead III
aVR(PE)	the amplitude of the ending of P wave in Lead aVR
aVL(PE)	the amplitude of the ending of P wave in Lead aVL
aVF(PE)	the amplitude of the ending of P wave in Lead aVF
V1(PE)	the amplitude of the ending of P wave in Lead V1
V2(PE)	the amplitude of the ending of P wave in Lead V2
V3(PE)	the amplitude of the ending of P wave in Lead V3
V4(PE)	the amplitude of the ending of P wave in Lead V4
V5(PE)	the amplitude of the ending of P wave in Lead V5
V6(PE)	the amplitude of the ending of P wave in Lead V6
I (Q)	the QRS complex amplitude in Lead I
II(Q)	the QRS complex amplitude in Lead II
III(Q)	the QRS complex amplitude in Lead III
aVR(Q)	the QRS complex amplitude in Lead aVR
aVL(Q)	the QRS complex amplitude in Lead aVL
aVF(Q)	the QRS complex amplitude in Lead aVF
V1(Q)	the QRS complex amplitude in Lead V1
V2(Q)	the QRS complex amplitude in Lead V2
V3(Q)	the QRS complex amplitude in Lead V3
V4(Q)	the QRS complex amplitude in Lead V4
V5(Q)	the QRS complex amplitude in Lead V5
V6(Q)	the QRS complex amplitude in Lead V6
I (R)	the QRS complex amplitude in Lead I
II(R)	the QRS complex amplitude in Lead II
III(R)	the QRS complex amplitude in Lead III
aVR(R)	the QRS complex amplitude in Lead aVR

aVL(R)	the QRS complex amplitude in Lead aVL
aVF(R)	the QRS complex amplitude in Lead aVF
V1(R)	the QRS complex amplitude in Lead V1
V2(R)	the QRS complex amplitude in Lead V2
V3(R)	the QRS complex amplitude in Lead V3
V4(R)	the QRS complex amplitude in Lead V4
V5(R)	the QRS complex amplitude in Lead V5
V6(R)	the QRS complex amplitude in Lead V6
I (S)	the S wave amplitude in Lead I
II(S)	the S wave amplitude in Lead II
III(S)	the S wave amplitude in Lead III
aVR(S)	the S wave amplitude in Lead aVR
aVL(S)	the S wave amplitude in Lead aVL
aVF(S)	the S wave amplitude in Lead aVF
V1(S)	the S wave amplitude in Lead V1
V2(S)	the S wave amplitude in Lead V2
V3(S)	the S wave amplitude in Lead V3
V4(S)	the S wave amplitude in Lead V4
V5(S)	the S wave amplitude in Lead V5
V6(S)	the S wave amplitude in Lead V6
I (R')	the R' wave amplitude in Lead I
II(R')	the R' wave amplitude in Lead II
III(R')	the R' wave amplitude in Lead III
aVR(R')	the R' wave amplitude in Lead aVR
aVL(R')	the R' wave amplitude in Lead aVL
aVF(R')	the R' wave amplitude in Lead aVF
V1(R')	the R' wave amplitude in Lead V1
V2(R')	the R' wave amplitude in Lead V2
V3(R')	the R' wave amplitude in Lead V3
V4(R')	the R' wave amplitude in Lead V4
V5(R')	the R' wave amplitude in Lead V5
V6(R')	the R' wave amplitude in Lead V6
I (S')	the S' wave amplitude in Lead I
II(S')	the S' wave amplitude in Lead II
III(S')	the S' wave amplitude in Lead III
aVR(S')	the S' wave amplitude in Lead aVR
aVL(S')	the S' wave amplitude in Lead aVL
aVF(S')	the S' wave amplitude in Lead aVF
V1(S')	the S' wave amplitude in Lead V1
V2(S')	the S' wave amplitude in Lead V2
V3(S')	the S' wave amplitude in Lead V3
V4(S')	the S' wave amplitude in Lead V4
V5(S')	the S' wave amplitude in Lead V5
V6(S')	the S' wave amplitude in Lead V6

I (TB)	the amplitude of the beginning of T wave in Lead I
II(TB)	the amplitude of the beginning of T wave in Lead II
III(TB)	the amplitude of the beginning of T wave in Lead III
aVR(TB)	the amplitude of the beginning of T wave in Lead aVR
aVL(TB)	the amplitude of the beginning of T wave in Lead aVL
aVF(TB)	the amplitude of the beginning of T wave in Lead aVF
V1(TB)	the amplitude of the beginning of T wave in Lead V1
V2(TB)	the amplitude of the beginning of T wave in Lead V2
V3(TB)	the amplitude of the beginning of T wave in Lead V3
V4(TB)	the amplitude of the beginning of T wave in Lead V4
V5(TB)	the amplitude of the beginning of T wave in Lead V5
V6(TB)	the amplitude of the beginning of T wave in Lead V6
I (TE)	the amplitude of the end of T wave in Lead I
II(TE)	the amplitude of the end of T wave in Lead II
III(TE)	the amplitude of the end of T wave in Lead III
aVR(TE)	the amplitude of the end of T wave in Lead aVR
aVL(TE)	the amplitude of the end of T wave in Lead aVL
aVF(TE)	the amplitude of the end of T wave in Lead aVF
V1(TE)	the amplitude of the end of T wave in Lead V1
V2(TE)	the amplitude of the end of T wave in Lead V2
V3(TE)	the amplitude of the end of T wave in Lead V3
V4(TE)	the amplitude of the end of T wave in Lead V4
V5(TE)	the amplitude of the end of T wave in Lead V5
V6(TE)	the amplitude of the end of T wave in Lead V6
I (ST20)	the amplitude of ST segment at 20 ms from J point in Lead I
II(ST20)	the amplitude of ST segment at 20 ms from J point in Lead II
III(ST20)	the amplitude of ST segment at 20 ms from J point in Lead III
aVR(ST20)	the amplitude of ST segment at 20 ms from J point in Lead aVR
aVL(ST20)	the amplitude of ST segment at 20 ms from J point in Lead aVL
aVF(ST20)	the amplitude of ST segment at 20 ms from J point in Lead aVF
V1(ST20)	the amplitude of ST segment at 20 ms from J point in Lead V1
V2(ST20)	the amplitude of ST segment at 20 ms from J point in Lead V2
V3(ST20)	the amplitude of ST segment at 20 ms from J point in Lead V3
V4(ST20)	the amplitude of ST segment at 20 ms from J point in Lead V4
V5(ST20)	the amplitude of ST segment at 20 ms from J point in Lead V5
V6(ST20)	the amplitude of ST segment at 20 ms from J point in Lead V6
I (ST40)	the amplitude of ST segment at 40 ms from J point in Lead I
II(ST40)	the amplitude of ST segment at 40 ms from J point in Lead II
III(ST40)	the amplitude of ST segment at 40 ms from J point in Lead III
aVR(ST40)	the amplitude of ST segment at 40 ms from J point in Lead aVR
aVL(ST40)	the amplitude of ST segment at 40 ms from J point in Lead aVL
aVF(ST40)	the amplitude of ST segment at 40 ms from J point in Lead aVF
V1(ST40)	the amplitude of ST segment at 40 ms from J point in Lead V1
V2(ST40)	the amplitude of ST segment at 40 ms from J point in Lead V2

V3(ST40)	the amplitude of ST segment at 40 ms from J point in Lead V3
V4(ST40)	the amplitude of ST segment at 40 ms from J point in Lead V4
V5(ST40)	the amplitude of ST segment at 40 ms from J point in Lead V5
V6(ST40)	the amplitude of ST segment at 40 ms from J point in Lead V6
I (ST60)	the amplitude of ST segment at 60 ms from J point in Lead I
II(ST60)	the amplitude of ST segment at 60 ms from J point in Lead II
III(ST60)	the amplitude of ST segment at 60 ms from J point in Lead III
aVR(ST60)	the amplitude of ST segment at 60 ms from J point in Lead aVR
aVL(ST60)	the amplitude of ST segment at 60 ms from J point in Lead aVL
aVF(ST60)	the amplitude of ST segment at 60 ms from J point in Lead aVF
V1(ST60)	the amplitude of ST segment at 60 ms from J point in Lead V1
V2(ST60)	the amplitude of ST segment at 60 ms from J point in Lead V2
V3(ST60)	the amplitude of ST segment at 60 ms from J point in Lead V3
V4(ST60)	the amplitude of ST segment at 60 ms from J point in Lead V4
V5(ST60)	the amplitude of ST segment at 60 ms from J point in Lead V5
V6(ST60)	the amplitude of ST segment at 60 ms from J point in Lead V6
I (ST80)	the amplitude of ST segment at 80 ms from J point in Lead I
II(ST80)	the amplitude of ST segment at 80 ms from J point in Lead II
III(ST80)	the amplitude of ST segment at 80 ms from J point in Lead III
aVR(ST80)	the amplitude of ST segment at 80 ms from J point in Lead aVR
aVL(ST80)	the amplitude of ST segment at 80 ms from J point in Lead aVL
aVF(ST80)	the amplitude of ST segment at 80 ms from J point in Lead aVF
V1(ST80)	the amplitude of ST segment at 80 ms from J point in Lead V1
V2(ST80)	the amplitude of ST segment at 80 ms from J point in Lead V2
V3(ST80)	the amplitude of ST segment at 80 ms from J point in Lead V3
V4(ST80)	the amplitude of ST segment at 80 ms from J point in Lead V4
V5(ST80)	the amplitude of ST segment at 80 ms from J point in Lead V5
V6(ST80)	the amplitude of ST segment at 80 ms from J point in Lead V6

Supplementary Table S2 Baseline characteristics between patients with or without STEMI

	Ctrl	STEMI	*P* value
n	502	318	
Age(years)	55.1±12.4	60.6±12.8	0.000
Gender(female)	224(44.6%)	58(18.2%)	0.000
Diabetes mellitus	98(19.5%)	58(18.2%)	0.715
Hypertension	224(44.6%)	113(35.5%)	0.011
Chronic kidney disease	12(2.4%)	7(2.2%)	0.532
CVD family history	23(4.6%)	20(6.3%)	0.302
WBC(*$10^{9/L}$)	7.28±3.00	9.15±3.13	0.881
RBC(*109/L)	4.27±0.95	4.38±0.69	0.039
HGB(g/ml)	128.09±16.83	131.49±19.69	0.013
PLT(*109/L)	207.26±59.49	216.83±60.41	0.007
ALB(g/L)	39.36±4.33	38.71±4.67	0.028
GLB(g/L)	24.65±3.85	25.19±4.91	0.061
K(mmol/L)	3.57±0.40	3.73±0.49	0.000
Na(mmol/L)	137.4±4.6	137.9±4.5	0.014
Ca(mmol/L)	2.16±0.31	2.14±1.25	0.697
GLU(mmol/L)	6.69±2.99	7.27±2.98	0.020
BUN(mmol/L)	5.1±2.7	5.8±2.9	0.001
CREAT(mmol/L)	82.07±58.45	76.08±42.21	0.199
CHOL(mmol/L)	4.52±1.22	4.50±1.18	0.860
TG(mmol/L)	1.59±1.08	1.64±1.14	0.461
HDL(mmol/L)	1.12±0.32	1.01±0.30	0.000
LDL(mmol/L)	2.80±1.00	2.93±1.01	0.042
CK-MB(U/L)	6.49±4.71	32.52±58.25	0.000

Supplementary Table S3 ECG features between control and STEMI

	Ctrl	STEMI	*P* value
n	502	318	
Maxmum HR	69.37±12.62	71.74±17.17	0.034
Minimun HR	78.66±20.03	86.79±25.49	0.000
Average HR	73.17±12.52	78.28±17.48	0.000
PR(ms)	151.71±24.46	153.04±34.42	0.548
P wave(ms)	108.8±21.88	103.8±26.14	0.005
QRS complex(ms)	102.72±10.69	105.62±13.86	0.002
T wave(ms)	183.61±21.69	176.21±34.65	0.001
QT interval(ms)	386.24±32.41	394.61±43.89	0.004
QTc interval(ms)	423.81±26.27	445.64±35.58	0.000
QRS axis	43.23±38.11	29.72±66.89	0.001
RV1+SV5 mv	0.57±0.33	0.49±0.38	0.001
RV5+SV1 mv	2.31±0.71	1.75±0.89	0.000
R-PB(ms)	199.7±31.2	199.19±45.24	0.861
R-P(ms)	136.46±24.42	145.17±39.51	0.000
R-PE(ms)	91.59±15.22	96.62±27.51	0.003
R-Q(ms)	49.39±3.98	49.54±4.61	0.629
R-S(ms)	53.33±7.79	56.08±11.62	0.000
R-TB(ms)	153.24±23.11	168.85±32.54	0.000
R-T(ms)	264.23±39.58	267.8±40.34	0.214
R-TE(ms)	336.85±32.04	345.06±44.14	0.004
Q-P(ms)	88.42±20.14	101.06±29.55	0.000
S-T(ms)	210.9±39.82	211.72±41.91	0.781
Q-PB(ms)	151.81±24.34	155.7±27.15	0.038
S-TE(ms)	283.52±32.77	288.98±46.03	0.066
I (PB)(mV)	0.06±0.03	0.06±0.03	0.054
II(PB)mV	0.11±0.04	0.1±0.05	0.034
III(PB)mV	0.07±0.04	0.06±0.04	0.004
aVR(PB)mV	0±0	0±0	0.147
aVL(PB)mV	0.02±0.02	0.03±0.03	0.028
aVF(PB)mV	0.08±0.04	0.08±0.04	0.018
V1(PB)mV	0.04±0.03	0.03±0.03	0.002
V2(PB)mV	0.06±0.03	0.05±0.04	0.000
V3(PB)mV	0.06±0.03	0.05±0.03	0.000
V4(PB)mV	0.06±0.02	0.05±0.03	0.000
V5(PB)mV	0.06±0.02	0.05±0.03	0.000
V6(PB)mV	0.06±0.02	0.05±0.03	0.055
I (PE)mV	0±0	0±0.01	0.046
II(PE)mV	0±0	0±0.01	0.252
III(PE)mV	-0.01±0.02	-0.01±0.02	0.078

aVR(PE)mV	-0.08±0.03	-0.07±0.03	0.338
aVL(PE)mV	-0.02±0.02	-0.02±0.02	0.014
aVF(PE)mV	0±0.01	0±0.01	0.232
V1(PE)mV	-0.03±0.03	-0.04±0.03	0.000
V2(PE)mV	-0.01±0.02	-0.01±0.02	0.000
V3(PE)mV	0±0.01	-0.01±0.02	0.000
V4(PE)mV	0±0.01	0±0.01	0.000
V5(PE)mV	0±0.01	0±0.01	0.020
V6(PE)mV	0±0	0±0.01	0.337
I (Q)mV	-0.03±0.03	-0.03±0.09	0.443
II(Q)mV	-0.03±0.04	-0.09±0.12	0.000
III(Q)mV	-0.06±0.11	-0.26±0.32	0.000
aVR(Q)mV	-0.38±0.34	-0.26±0.28	0.000
aVL(Q)mV	-0.04±0.07	-0.04±0.11	0.944
aVF(Q)mV	-0.03±0.06	-0.15±0.19	0.000
V1(Q)mV	-0.04±0.2	-0.35±0.49	0.000
V2(Q)mV	-0.01±0.13	-0.51±0.85	0.000
V3(Q)mV	-0.01±0.14	-0.4±0.71	0.000
V4(Q)mV	-0.01±0.06	-0.16±0.36	0.000
V5(Q)mV	-0.02±0.04	-0.08±0.17	0.000
V6(Q)mV	-0.03±0.04	-0.05±0.08	0.000
I (R)mV	0.61±0.28	0.54±0.32	0.003
II(R)mV	0.77±0.32	0.42±0.3	0.000
III(R)mV	0.37±0.33	0.26±0.27	0.000
aVR(R)mV	0.07±0.08	0.08±0.1	0.168
aVL(R)mV	0.33±0.25	0.42±0.31	0.000
aVF(R)mV	0.52±0.33	0.29±0.27	0.000
V1(R)mV	0.23±0.18	0.15±0.24	0.000
V2(R)mV	0.66±0.35	0.46±0.58	0.000
V3(R)mV	1.01±0.5	0.69±0.8	0.000
V4(R)mV	1.52±0.61	0.96±0.92	0.000
V5(R)mV	1.49±0.56	0.98±0.75	0.000
V6(R)mV	1.2±0.45	0.81±0.56	0.000
I (S)mV	-0.1±0.11	-0.1±0.13	0.682
II(S)mV	-0.09±0.13	-0.09±0.14	0.866
III(S)mV	-0.17±0.24	-0.13±0.24	0.007
aVR(S)mV	-0.29±0.39	-0.19±0.27	0.000
aVL(S)mV	-0.15±0.16	-0.12±0.16	0.016
aVF(S)mV	-0.1±0.15	-0.08±0.15	0.158
V1(S)mV	-0.83±0.39	-0.78±0.47	0.166
V2(S)mV	-1.23±0.6	-0.85±0.8	0.000
V3(S)mV	-0.91±0.6	-0.67±0.63	0.000
V4(S)mV	-0.61±0.47	-0.52±0.45	0.006
V5(S)mV	-0.35±0.3	-0.34±0.29	0.518

V6(S)mV	-0.18±0.19	-0.15±0.18	0.041
I (R')mV	0±0	0±0.02	0.312
II(R')mV	0±0.01	0.01±0.03	0.046
III(R')mV	0.05±0.11	0.02±0.06	0.000
aVR(R')mV	0.02±0.05	0.03±0.07	0.001
aVL(R')mV	0.01±0.02	0±0.02	0.037
aVF(R')mV	0.02±0.06	0.02±0.06	0.991
V1(R')mV	0.02±0.13	0.01±0.06	0.077
V2(R')mV	0±0.01	0±0.02	0.216
V3(R')mV	0±0.03	0±0.05	0.682
V4(R')mV	0±0.01	0.01±0.06	0.277
V5(R')mV	0±0.01	0±0	0.059
V6(R')mV	0±0	0±0.01	0.658
I (S')mV	0±0	0±0	0.318
II(S')mV	0±0	0±0.02	0.159
III(S')mV	-0.01±0.04	0±0.03	0.150
aVR(S')mV	0±0	0±0	0.318
aVL(S')mV	0±0.01	0±0	0.336
aVF(S')mV	0±0.02	-0.01±0.04	0.187
V1(S')mV	0±0.02	0±0	0.037
V2(S')mV	0±0.02	-0.01±0.11	0.226
V3(S')mV	0±0	0±0.05	0.272
V4(S')mV	0±0.01	-0.01±0.07	0.084
V5(S')mV	0±0	0±0	0.000
V6(S')mV	0±0	0±0.01	0.318
I (TB)mV	0.13±0.07	0.08±0.08	0.000
II(TB)mV	0.16±0.08	0.09±0.09	0.000
III(TB)mV	0.06±0.06	0.07±0.09	0.009
aVR(TB)mV	0±0.01	0.01±0.03	0.000
aVL(TB)mV	0.06±0.06	0.08±0.09	0.011
aVF(TB)mV	0.1±0.07	0.08±0.09	0.000
V1(TB)mV	0.06±0.09	0.08±0.1	0.001
V2(TB)mV	0.33±0.21	0.29±0.27	0.058
V3(TB)mV	0.32±0.21	0.23±0.25	0.000
V4(TB)mV	0.31±0.2	0.15±0.19	0.000
V5(TB)mV	0.26±0.15	0.1±0.12	0.000
V6(TB)mV	0.2±0.11	0.08±0.1	0.000
I (TE)mV	0.13±0.07	0.08±0.08	0.000
II(TE)mV	0.16±0.08	0.09±0.09	0.000
III(TE)mV	0.06±0.06	0.07±0.09	0.009
aVR(TE)mV	0±0.01	0.01±0.03	0.000
aVL(TE)mV	0.06±0.06	0.08±0.09	0.011
aVF(TE)mV	0.1±0.07	0.08±0.09	0.000
V1(TE)mV	0.06±0.09	0.08±0.1	0.001

V2(TE)mV	0.33±0.21	0.29±0.27	0.058
V3(TE)mV	0.32±0.21	0.23±0.25	0.000
V4(TE)mV	0.31±0.2	0.15±0.19	0.000
V5(TE)mV	0.26±0.15	0.1±0.12	0.000
V6(TE)mV	0.2±0.11	0.08±0.1	0.000
I (ST20)mV	0±0.02	-0.01±0.05	0.000
II(ST20)mV	0.01±0.03	0.03±0.08	0.000
III(ST20)mV	0±0.02	0.04±0.11	0.000
aVR(ST20)mV	0±0.02	-0.01±0.04	0.146
aVL(ST20)mV	0±0.01	-0.02±0.07	0.000
aVF(ST20)mV	0±0.02	0.03±0.09	0.000
V1(ST20)mV	0.04±0.04	0.05±0.09	0.039
V2(ST20)mV	0.09±0.07	0.11±0.16	0.016
V3(ST20)mV	0.06±0.06	0.08±0.16	0.008
V4(ST20)mV	0.02±0.05	0.04±0.13	0.089
V5(ST20)mV	0±0.04	0.01±0.09	0.390
V6(ST20)mV	0±0.03	0±0.06	0.482
I (ST40)mV	0.01±0.02	-0.01±0.05	0.000
II(ST40)mV	0.01±0.03	0.03±0.08	0.000
III(ST40)mV	0±0.02	0.04±0.11	0.000
aVR(ST40)mV	-0.01±0.02	-0.01±0.04	0.230
aVL(ST40)mV	0±0.02	-0.02±0.07	0.000
aVF(ST40)mV	0.01±0.03	0.03±0.09	0.000
V1(ST40)mV	0.05±0.04	0.06±0.08	0.123
V2(ST40)mV	0.12±0.08	0.14±0.16	0.025
V3(ST40)mV	0.09±0.08	0.11±0.17	0.011
V4(ST40)mV	0.05±0.06	0.06±0.14	0.172
V5(ST40)mV	0.02±0.04	0.02±0.09	0.911
V6(ST40)mV	0.01±0.03	0.01±0.06	0.889
I (ST60)mV	0.02±0.03	0±0.05	0.000
II(ST60)mV	0.02±0.03	0.04±0.08	0.000
III(ST60)mV	0±0.03	0.04±0.11	0.000
aVR(ST60)mV	-0.02±0.03	-0.02±0.04	0.783
aVL(ST60)mV	0.01±0.02	-0.02±0.07	0.000
aVF(ST60)mV	0.01±0.03	0.04±0.09	0.000
V1(ST60)mV	0.06±0.05	0.07±0.08	0.220
V2(ST60)mV	0.16±0.1	0.18±0.18	0.050
V3(ST60)mV	0.12±0.1	0.14±0.18	0.070
V4(ST60)mV	0.07±0.07	0.08±0.15	0.933
V5(ST60)mV	0.04±0.05	0.03±0.1	0.115
V6(ST60)mV	0.02±0.04	0.01±0.07	0.146
I (ST80)mV	0.03±0.03	0.01±0.06	0.000
II(ST80)mV	0.04±0.04	0.05±0.09	0.004
III(ST80)mV	0±0.03	0.04±0.11	0.000

aVR(ST80)mV	-0.03±0.03	-0.03±0.05	0.173
aVL(ST80)mV	0.01±0.02	-0.02±0.08	0.000
aVF(ST80)mV	0.02±0.03	0.05±0.1	0.000
V1(ST80)mV	0.07±0.07	0.08±0.09	0.183
V2(ST80)mV	0.2±0.13	0.22±0.19	0.127
V3(ST80)mV	0.17±0.13	0.17±0.19	0.541
V4(ST80)mV	0.11±0.1	0.09±0.15	0.044
V5(ST80)mV	0.07±0.07	0.04±0.11	0.000
V6(ST80)mV	0.04±0.05	0.02±0.08	0.000

Supplementary Table S4 ECG features between Cohort 1 and Cohort 2

	Cohort 1	Cohort 2	*P* value
n	736	90	
Minimun HR	70.54±14.55	68.12±14.89	0.145
Maxmum HR	82.12±23.01	79.15±19.08	0.250
Average HR	75.43±14.97	72.73±13.52	0.110
PR(ms)	152.83±28.45	147.1±30.64	0.081
P wave(ms)	107.59±23.57	100.64±24.33	0.010
QRS(ms)	103.79±12.44	104.34±8.57	0.690
T wave(ms)	180.72±27.05	180.98±32.65	0.934
QT interval(ms)	388.6±37.34	396.99±38.04	0.050
QTc interval(ms)	432.13±32.39	433.53±28.89	0.700
QRS axis	39.32±51.92	26.63±47.66	0.031
RV1+SV5 mv	0.54±0.36	0.51±0.33	0.407
RV5+SV1 mv	2.1±0.83	2.05±0.83	0.607
R-PB(ms)	199.86±37.58	196.44±34.44	0.421
R-P(ms)	139.57±31.63	142.14±29.66	0.473
R-PE(ms)	93.27±20.75	95.8±22.95	0.291
R-Q(ms)	49.4±4.31	49.87±3.52	0.330
R-S(ms)	54.39±9.86	54.47±6.39	0.942
R-TB(ms)	158.49±28.31	166.14±26.2	0.017
R-T(ms)	265.36±39.55	267.77±42.84	0.596
R-TE(ms)	339.2±37.17	347.12±38.73	0.063
Q-P(ms)	93.22±25.06	94.2±24.41	0.731
S-T(ms)	210.97±40.37	213.3±42.9	0.615
Q-PB(ms)	153.86±25.42	148.73±26.12	0.078
S-TE(ms)	284.82±38.32	292.65±39.76	0.074
I (PB)mV	0.06±0.03	0.06±0.03	0.841
II(PB)mV	0.1±0.04	0.09±0.04	0.051
III(PB)mV	0.07±0.04	0.06±0.04	0.036
aVR(PB)mV	0±0	0±0	0.403
aVL(PB)mV	0.03±0.02	0.03±0.03	0.339
aVF(PB)mV	0.08±0.04	0.07±0.04	0.055
V1(PB)mV	0.04±0.03	0.03±0.03	0.388
V2(PB)mV	0.05±0.03	0.05±0.03	0.199
V3(PB)mV	0.06±0.03	0.06±0.03	0.768
V4(PB)mV	0.06±0.03	0.06±0.02	0.524
V5(PB)mV	0.06±0.02	0.06±0.03	0.757
V6(PB)mV	0.06±0.02	0.06±0.02	0.640
I (PE)mV	0±0	0±0.01	0.445
II(PE)mV	0±0.01	0±0	0.778
III(PE)mV	-0.01±0.02	-0.01±0.02	0.245
aVR(PE)mV	-0.08±0.03	-0.07±0.03	0.225
aVL(PE)mV	-0.02±0.02	-0.02±0.02	0.439

aVF(PE)mV	0±0.01	0±0.01	0.696
V1(PE)mV	-0.03±0.03	-0.04±0.03	0.216
V2(PE)mV	-0.01±0.02	-0.01±0.02	0.613
V3(PE)mV	0±0.01	0±0.02	0.971
V4(PE)mV	0±0.01	0±0.01	0.606
V5(PE)mV	0±0.01	0±0.01	0.901
V6(PE)mV	0±0.01	0±0.01	0.905
I (Q)mV	-0.03±0.07	-0.02±0.03	0.230
II(Q)mV	-0.05±0.08	-0.07±0.11	0.036
III(Q)mV	-0.13±0.23	-0.22±0.29	0.005
aVR(Q)mV	-0.34±0.33	-0.29±0.29	0.205
aVL(Q)mV	-0.04±0.09	-0.03±0.06	0.228
aVF(Q)mV	-0.07±0.13	-0.12±0.18	0.001
V1(Q)mV	-0.15±0.37	-0.23±0.4	0.080
V2(Q)mV	-0.19±0.58	-0.37±0.69	0.007
V3(Q)mV	-0.15±0.49	-0.24±0.51	0.121
V4(Q)mV	-0.07±0.24	-0.08±0.26	0.647
V5(Q)mV	-0.05±0.12	-0.03±0.08	0.303
V6(Q)mV	-0.04±0.06	-0.03±0.05	0.645
I (R)mV	0.58±0.3	0.56±0.26	0.465
II(R)mV	0.65±0.36	0.49±0.29	0.000
III(R)mV	0.33±0.32	0.24±0.24	0.009
aVR(R)mV	0.08±0.09	0.07±0.07	0.256
aVL(R)mV	0.36±0.28	0.39±0.24	0.339
aVF(R)mV	0.44±0.34	0.31±0.26	0.000
V1(R)mV	0.2±0.21	0.15±0.18	0.014
V2(R)mV	0.58±0.45	0.54±0.54	0.379
V3(R)mV	0.89±0.64	0.8±0.74	0.221
V4(R)mV	1.32±0.78	1.17±0.86	0.118
V5(R)mV	1.29±0.69	1.29±0.7	0.908
V6(R)mV	1.04±0.53	1.12±0.56	0.220
I (S)mV	-0.1±0.12	-0.1±0.1	0.905
II(S)mV	-0.1±0.14	-0.05±0.12	0.003
III(S)mV	-0.16±0.24	-0.09±0.19	0.008
aVR(S)mV	-0.25±0.36	-0.21±0.29	0.268
aVL(S)mV	-0.14±0.16	-0.13±0.14	0.557
aVF(S)mV	-0.1±0.15	-0.05±0.12	0.003
V1(S)mV	-0.82±0.42	-0.77±0.48	0.362
V2(S)mV	-1.1±0.7	-0.88±0.73	0.006
V3(S)mV	-0.82±0.62	-0.78±0.65	0.583
V4(S)mV	-0.57±0.47	-0.61±0.44	0.549
V5(S)mV	-0.34±0.3	-0.37±0.27	0.512
V6(S)mV	-0.16±0.19	-0.17±0.18	0.630
I (R')mV	0±0.01	0±0	0.666

II(R')mV	0±0.02	0.01±0.03	0.405
III(R')mV	0.04±0.1	0.03±0.08	0.603
aVR(R')mV	0.02±0.06	0.01±0.04	0.161
aVL(R')mV	0.01±0.02	0±0.01	0.059
aVF(R')mV	0.02±0.06	0.01±0.05	0.577
V1(R')mV	0.02±0.11	0±0.02	0.287
V2(R')mV	0±0.02	0±0.01	0.683
V3(R')mV	0±0.03	0.01±0.09	0.476
V4(R')mV	0±0.04	0±0.02	0.843
V5(R')mV	0±0.01	0±0	0.518
V6(R')mV	0±0.01	0±0	0.571
I (S')mV	0±0	0±0	0.732
II(S')mV	0±0.01	0±0	0.629
III(S')mV	-0.01±0.03	0±0	0.141
aVR(S')mV	0±0	0±0	0.732
aVL(S')mV	0±0.01	0±0	0.649
aVF(S')mV	0±0.04	0±0	0.264
V1(S')mV	0±0.02	0±0.01	0.820
V2(S')mV	0±0.08	0±0	0.605
V3(S')mV	0±0.01	-0.01±0.09	0.006
V4(S')mV	0±0.04	-0.01±0.06	0.432
V5(S')mV	0±0	0±0	0.000
V6(S')mV	0±0	0±0	0.732
I (TB)mV	0.12±0.08	0.1±0.08	0.110
II(TB)mV	0.14±0.09	0.09±0.08	0.000
III(TB)mV	0.06±0.08	0.05±0.08	0.192
aVR(TB)mV	0±0.02	0.01±0.02	0.003
aVL(TB)mV	0.06±0.07	0.09±0.1	0.011
aVF(TB)mV	0.09±0.08	0.06±0.08	0.001
V1(TB)mV	0.07±0.09	0.07±0.11	0.917
V2(TB)mV	0.31±0.23	0.3±0.29	0.636
V3(TB)mV	0.28±0.23	0.26±0.26	0.459
V4(TB)mV	0.25±0.21	0.2±0.21	0.039
V5(TB)mV	0.2±0.16	0.15±0.16	0.006
V6(TB)mV	0.16±0.12	0.12±0.13	0.006
I (TE)mV	0.12±0.08	0.1±0.08	0.110
II(TE)mV	0.14±0.09	0.09±0.08	0.000
III(TE)mV	0.06±0.08	0.05±0.08	0.192
aVR(TE)mV	0±0.02	0.01±0.02	0.058
aVL(TE)mV	0.06±0.07	0.09±0.1	0.039
aVF(TE)mV	0.09±0.08	0.06±0.08	0.001
V1(TE)mV	0.07±0.09	0.07±0.11	0.917
V2(TE)mV	0.31±0.23	0.3±0.29	0.636
V3(TE)mV	0.28±0.23	0.26±0.26	0.459

V4(TE)mV	0.25±0.21	0.2±0.21	0.039
V5(TE)mV	0.2±0.16	0.15±0.16	0.006
V6(TE)mV	0.16±0.12	0.12±0.13	0.006
I (ST20)mV	0±0.03	-0.02±0.05	0.000
II(ST20)mV	0.01±0.06	0.02±0.05	0.414
III(ST20)mV	0.01±0.07	0.03±0.08	0.027
aVR(ST20)mV	-0.01±0.03	0±0.02	0.203
aVL(ST20)mV	-0.01±0.04	-0.02±0.06	0.004
aVF(ST20)mV	0.01±0.06	0.02±0.06	0.106
V1(ST20)mV	0.04±0.05	0.05±0.1	0.166
V2(ST20)mV	0.1±0.11	0.08±0.12	0.356
V3(ST20)mV	0.07±0.11	0.06±0.12	0.691
V4(ST20)mV	0.03±0.09	0.02±0.11	0.646
V5(ST20)mV	0.01±0.06	0±0.07	0.910
V6(ST20)mV	0±0.04	-0.01±0.05	0.201
I (ST40)mV	0.01±0.03	-0.01±0.05	0.000
II(ST40)mV	0.02±0.06	0.02±0.05	0.550
III(ST40)mV	0.01±0.07	0.03±0.09	0.036
aVR(ST40)mV	-0.01±0.03	-0.01±0.02	0.118
aVL(ST40)mV	0±0.04	-0.02±0.06	0.005
aVF(ST40)mV	0.02±0.06	0.03±0.07	0.154
V1(ST40)mV	0.06±0.06	0.06±0.06	0.439
V2(ST40)mV	0.13±0.12	0.12±0.12	0.581
V3(ST40)mV	0.1±0.12	0.1±0.13	0.911
V4(ST40)mV	0.05±0.09	0.05±0.11	0.964
V5(ST40)mV	0.02±0.06	0.02±0.08	0.691
V6(ST40)mV	0.01±0.05	0±0.05	0.372
I (ST60)mV	0.01±0.04	0±0.05	0.001
II(ST60)mV	0.03±0.06	0.03±0.06	0.862
III(ST60)mV	0.02±0.07	0.03±0.1	0.065
aVR(ST60)mV	-0.02±0.03	-0.01±0.03	0.068
aVL(ST60)mV	0±0.05	-0.02±0.07	0.015
aVF(ST60)mV	0.02±0.06	0.03±0.08	0.262
V1(ST60)mV	0.06±0.07	0.07±0.06	0.593
V2(ST60)mV	0.16±0.14	0.16±0.14	0.608
V3(ST60)mV	0.13±0.13	0.13±0.15	0.811
V4(ST60)mV	0.07±0.1	0.07±0.12	0.991
V5(ST60)mV	0.03±0.07	0.04±0.09	0.584
V6(ST60)mV	0.02±0.05	0.01±0.06	0.495
I (ST80)mV	0.02±0.04	0.01±0.05	0.003
II(ST80)mV	0.04±0.06	0.04±0.07	0.594
III(ST80)mV	0.02±0.07	0.03±0.11	0.192
aVR(ST80)mV	-0.03±0.04	-0.02±0.03	0.030
aVL(ST80)mV	0±0.05	-0.01±0.08	0.038

aVF(ST80)mV	0.03±0.06	0.03±0.08	0.633
V1(ST80)mV	0.07±0.08	0.08±0.08	0.650
V2(ST80)mV	0.21±0.16	0.21±0.16	0.706
V3(ST80)mV	0.17±0.15	0.17±0.17	0.856
V4(ST80)mV	0.11±0.12	0.1±0.14	0.841
V5(ST80)mV	0.06±0.09	0.06±0.1	0.700
V6(ST80)mV	0.03±0.06	0.03±0.07	0.521

Supplementary Table S5 ECG features among different locations of infarct-related arteries.

	Ctrl	LAD	RCA	LCX	*P* value
n	502	158	115	45	
Minimum HR	151.71±12.62	151.37±17.54	155.27±16.83	153.22±16.98	0.107
Miaxmum HR	108.8±20.03	103.93±25.62	102.89±25.48	105.67±25.57	0.000
Average HR	102.72±12.52	106.99±17.32	105.71±17.81	100.6±17.3	0.000
PR(ms)	151.71±24.459	151.37±34.446	155.27±34.281	153.22±35.158	0.649
P 波(ms)	108.8±21.88	103.93±27.323	102.89±25.824	105.67±22.887	0.028
QRS(ms)	102.72±10.689	106.99±12.464	105.71±16.883	100.6±7.608	0.000
T 波(ms)	183.61±21.694	165.11±32.862	186.87±30.704	187.98±38.36	0.000
QT(ms)	386.24±32.409	390.7±44.52	396.9±40.256	402.49±49.73	0.003
QTc(ms)	423.81±26.269	443.87±37.137	447.42±32.807	447.31±37.289	0.000
QRS axis	43.23±38.111	44.17±74.134	8.31±54.242	33.71±55.094	0.000
RV1+SV5 mv	0.57±0.33105	0.44±0.39471	0.52±0.37279	0.57±0.3195	0.000
RV5+SV1 mv	2.31±0.70923	1.56±0.83317	1.91±0.9146	2.05±0.88383	0.000
R-PB(ms)	199.7±31.20219	198.33±45.1145	200.08±47.62484	199.96±40.01815	0.977
R-P(ms)	136.46±24.42172	144.14±38.52577	148.21±41.88529	141.04±36.86397	0.001
R-PE(ms)	91.59±15.21501	95.75±24.14887	98.73±30.88897	94.29±29.68135	0.004
R-Q(ms)	49.39±3.97991	50.44±4.83674	49.01±4.15805	47.78±4.25809	0.001
R-S(ms)	53.33±7.79457	56.55±9.85923	56.7±15.08861	52.82±4.86775	0.000
R-TB(ms)	153.24±23.10517	175.15±30.32881	161.02±31.85906	166.73±37.51085	0.000
R-T(ms)	264.23±39.57619	261.19±36.48089	275.35±43.89682	271.69±40.76754	0.015
R-TE(ms)	336.85±32.03638	340.26±44.59873	347.89±40.92436	354.71±49.01791	0.001
Q-P(ms)	88.42±20.13627	99.27±28.61393	105.13±30.33165	96.98±30.20949	0.000
S-T(ms)	210.9±39.81739	204.64±37.73275	218.64±46.21141	218.87±41.03524	0.022
Q-PB(ms)	151.81±24.34498	154.09±27.2386	157.68±27.40231	156.33±26.39301	0.118
S-TE(ms)	283.52±32.76607	283.71±46.33144	291.18±43.6217	301.89±48.91097	0.006
I (PB)mV	0.06±0.02626	0.06±0.02996	0.06±0.03002	0.07±0.02634	0.017
II(PB)mV	0.11±0.04334	0.1±0.04573	0.09±0.04426	0.11±0.04057	0.002
III(PB)mV	0.07±0.04248	0.06±0.04033	0.06±0.03361	0.07±0.03811	0.009
aVR(PB)mV	0±0.00134	0±0.00318	0±0.00656	0±0	0.072
aVL(PB)mV	0.02±0.02382	0.03±0.02801	0.02±0.02607	0.02±0.02483	0.011
aVF(PB)mV	0.08±0.0428	0.08±0.04124	0.07±0.03815	0.09±0.03753	0.006
V1(PB)mV	0.04±0.02649	0.04±0.0308	0.03±0.02836	0.04±0.02756	0.000
V2(PB)mV	0.06±0.02789	0.05±0.04276	0.04±0.03397	0.06±0.03136	0.000
V3(PB)mV	0.06±0.02538	0.06±0.03202	0.05±0.03037	0.06±0.02885	0.000
V4(PB)mV	0.06±0.02363	0.06±0.0298	0.05±0.02727	0.06±0.03096	0.000
V5(PB)mV	0.06±0.02251	0.06±0.02829	0.05±0.02629	0.06±0.02803	0.000
V6(PB)mV	0.06±0.02214	0.05±0.02821	0.05±0.02686	0.06±0.02423	0.000
I (PE)mV	0±0.00161	0±0.00636	0±0.00725	0±0	0.045
II(PE)mV	0±0.00385	0±0.00554	0±0.01179	0±0	0.152
III(PE)mV	-0.01±0.01895	-0.01±0.02404	-0.01±0.02279	-0.01±0.01792	0.027

aVR(PE)mV	-0.08±0.02702	-0.08±0.03133	-0.07±0.03279	-0.08±0.02742	0.005
aVL(PE)mV	-0.02±0.02301	-0.02±0.02124	-0.02±0.0192	-0.02±0.02109	0.111
aVF(PE)mV	0±0.00668	0±0.00967	0±0.01327	0±0.00447	0.375
V1(PE)mV	-0.03±0.02887	-0.04±0.03113	-0.04±0.03219	-0.05±0.03655	0.000
V2(PE)mV	-0.01±0.01611	-0.01±0.02177	-0.01±0.02516	-0.01±0.01992	0.000
V3(PE)mV	0±0.00802	0±0.01681	-0.01±0.02157	0±0.01338	0.000
V4(PE)mV	0±0.0059	0±0.01285	0±0.0146	0±0.01041	0.001
V5(PE)mV	0±0.00507	0±0.01142	0±0.01122	0±0.00625	0.041
V6(PE)mV	0±0.00381	0±0.00841	0±0.0056	0±0	0.208
I (Q)mV	-0.03±0.03208	-0.03±0.05448	-0.03±0.13977	-0.03±0.04251	0.789
II(Q)mV	-0.03±0.03987	-0.05±0.09288	-0.14±0.13458	-0.08±0.09537	0.000
III(Q)mV	-0.06±0.10919	-0.12±0.21261	-0.48±0.36453	-0.2±0.21777	0.000
aVR(Q)mV	-0.38±0.34138	-0.31±0.26821	-0.22±0.27712	-0.17±0.28435	0.000
aVL(Q)mV	-0.04±0.07001	-0.06±0.09238	-0.02±0.1404	-0.04±0.04873	0.008
aVF(Q)mV	-0.03±0.06039	-0.07±0.12418	-0.26±0.21815	-0.12±0.12985	0.000
V1(Q)mV	-0.04±0.20244	-0.62±0.52689	-0.09±0.25586	-0.04±0.17309	0.000
V2(Q)mV	-0.01±0.13048	-1±0.97785	-0.03±0.18575	-0.05±0.20961	0.000
V3(Q)mV	-0.01±0.14134	-0.77±0.84845	-0.02±0.07485	-0.06±0.23337	0.000
V4(Q)mV	-0.01±0.06311	-0.29±0.47648	-0.02±0.06166	-0.04±0.09264	0.000
V5(Q)mV	-0.02±0.04189	-0.11±0.22106	-0.05±0.10899	-0.06±0.07475	0.000
V6(Q)mV	-0.03±0.03648	-0.04±0.07754	-0.05±0.07901	-0.07±0.06629	0.000
I (R)mV	0.61±0.27968	0.44±0.27271	0.66±0.34156	0.61±0.30686	0.000
II(R)mV	0.77±0.3222	0.46±0.30409	0.36±0.28686	0.47±0.28119	0.000
III(R)mV	0.37±0.32637	0.26±0.25519	0.25±0.30608	0.26±0.22748	0.000
aVR(R)mV	0.07±0.08458	0.08±0.09813	0.09±0.11408	0.07±0.06471	0.402
aVL(R)mV	0.33±0.25499	0.32±0.25475	0.57±0.32942	0.44±0.28063	0.000
aVF(R)mV	0.52±0.33416	0.32±0.27229	0.24±0.27844	0.29±0.24121	0.000
V1(R)mV	0.23±0.18451	0.07±0.21345	0.21±0.22782	0.29±0.23648	0.000
V2(R)mV	0.66±0.34775	0.09±0.21447	0.81±0.57149	0.87±0.62555	0.000
V3(R)mV	1.01±0.50292	0.18±0.38627	1.12±0.75047	1.35±0.84681	0.000
V4(R)mV	1.52±0.60538	0.41±0.57515	1.48±0.90497	1.58±0.81401	0.000
V5(R)mV	1.49±0.55904	0.66±0.59164	1.3±0.81119	1.28±0.61267	0.000
V6(R)mV	1.2±0.45043	0.7±0.50384	0.91±0.62855	0.96±0.52454	0.000
I (S)mV	183.61±0.11	165.11±0.11	186.87±0.15	187.98±0.13	0.341
II(S)mV	386.24±0.13	390.7±0.15	396.9±0.13	402.49±0.12	0.220
III(S)mV	423.81±0.24	443.87±0.26	447.42±0.16	447.31±0.29	0.000
aVR(S)mV	43.23±0.39	44.17±0.21	8.31±0.29	33.71±0.31	0.000
aVL(S)mV	0.57±0.16	0.44±0.14	0.52±0.18	0.57±0.16	0.005
aVF(S)mV	2.31±0.15	1.56±0.18	1.91±0.1	2.05±0.14	0.000
V1(S)mV	199.7±0.39	198.33±0.47	200.08±0.38	199.96±0.53	0.000
V2(S)mV	136.46±0.6	144.14±0.93	148.21±0.57	141.04±0.68	0.000
V3(S)mV	91.59±0.6	95.75±0.7	98.73±0.49	94.29±0.57	0.000
V4(S)mV	49.39±0.47	50.44±0.5	49.01±0.41	47.78±0.39	0.032
V5(S)mV	53.33±0.3	56.55±0.31	56.7±0.28	52.82±0.23	0.238

V6(S)mV	153.24±0.19	175.15±0.19	161.02±0.18	166.73±0.15	0.218
I (R')mV	264.23±0	261.19±0	275.35±0.03	271.69±0	0.066
II(R')mV	336.85±0.01	340.26±0.04	347.89±0.02	354.71±0.05	0.033
III(R')mV	88.42±0.11	99.27±0.08	105.13±0.03	96.98±0.05	0.000
aVR(R')mV	210.9±0.05	204.64±0.06	218.64±0.08	218.87±0.09	0.000
aVL(R')mV	151.81±0.02	154.09±0.02	157.68±0.02	156.33±0.01	0.216
aVF(R')mV	283.52±0.06	283.71±0.07	291.18±0.03	301.89±0.05	0.090
V1(R')mV	0.06±0.13	0.06±0.05	0.06±0.05	0.07±0.09	0.447
V2(R')mV	0.11±0.01	0.1±0	0.09±0.04	0.11±0.01	0.004
V3(R')mV	0.07±0.03	0.06±0.07	0.06±0.01	0.07±0.06	0.717
V4(R')mV	0±0.01	0±0.08	0±0	0±0.04	0.189
V5(R')mV	0.02±0.01	0.03±0	0.02±0	0.02±0	0.521
V6(R')mV	0.08±0	0.08±0.01	0.07±0	0.09±0	0.498
I (S')mV	0.04±0	0.04±0	0.03±0	0.04±0	0.889
II(S')mV	0.06±0	0.05±0.02	0.04±0	0.06±0	0.039
III(S')mV	0.06±0.04	0.06±0.04	0.05±0	0.06±0	0.178
aVR(S')mV	0.06±0	0.06±0	0.05±0	0.06±0	0.889
aVL(S')mV	0.06±0.01	0.06±0	0.05±0	0.06±0	0.826
aVF(S')mV	0.06±0.02	0.05±0.06	0.05±0	0.06±0	0.006
V1(S')mV	0±0.02	0±0	0±0	0±0	0.430
V2(S')mV	0±0.02	0±0	0±0.04	0±0.29	0.001
V3(S')mV	-0.01±0	-0.01±0.07	-0.01±0.01	-0.01±0	0.307
V4(S')mV	-0.08±0.01	-0.08±0.1	-0.07±0.03	-0.08±0	0.021
V5(S')mV	-0.02±0	-0.02±0	-0.02±0	-0.02±0	0.000
V6(S')mV	0±0	0±0.01	0±0	0±0	0.242
I (TB)mV	-0.03±0.07	-0.04±0.07	-0.04±0.08	-0.05±0.08	0.000
II(TB)mV	-0.01±0.08	-0.01±0.09	-0.01±0.08	-0.01±0.07	0.000
III(TB)mV	0±0.06	0±0.1	-0.01±0.08	0±0.07	0.000
aVR(TB)mV	0±0.01	0±0.03	0±0.03	0±0.03	0.000
aVL(TB)mV	0±0.06	0±0.05	0±0.1	0±0.08	0.000
aVF(TB)mV	0±0.07	0±0.09	0±0.08	0±0.07	0.000
V1(TB)mV	-0.03±0.09	-0.03±0.08	-0.03±0.09	-0.03±0.14	0.000
V2(TB)mV	-0.03±0.21	-0.05±0.16	-0.14±0.25	-0.08±0.33	0.000
V3(TB)mV	-0.06±0.21	-0.12±0.14	-0.48±0.26	-0.2±0.32	0.000
V4(TB)mV	-0.38±0.2	-0.31±0.12	-0.22±0.21	-0.17±0.24	0.000
V5(TB)mV	-0.04±0.15	-0.06±0.1	-0.02±0.13	-0.04±0.13	0.000
V6(TB)mV	-0.03±0.11	-0.07±0.09	-0.26±0.1	-0.12±0.11	0.000
I (TE)mV	-0.04±0.07	-0.62±0.07	-0.09±0.08	-0.04±0.08	0.000
II(TE)mV	-0.01±0.08	-1±0.09	-0.03±0.08	-0.05±0.07	0.000
III(TE)mV	-0.01±0.06	-0.77±0.1	-0.02±0.08	-0.06±0.07	0.000
aVR(TE)mV	-0.01±0.01	-0.29±0.03	-0.02±0.03	-0.04±0.03	0.000
aVL(TE)mV	-0.02±0.06	-0.11±0.05	-0.05±0.1	-0.06±0.08	0.000
aVF(TE)mV	-0.03±0.07	-0.04±0.09	-0.05±0.08	-0.07±0.07	0.000
V1(TE)mV	0.61±0.09	0.44±0.08	0.66±0.09	0.61±0.14	0.000

V2(TE)mV	0.77±0.21	0.46±0.16	0.36±0.25	0.47±0.33	0.000
V3(TE)mV	0.37±0.21	0.26±0.14	0.25±0.26	0.26±0.32	0.000
V4(TE)mV	0.07±0.2	0.08±0.12	0.09±0.21	0.07±0.24	0.000
V5(TE)mV	0.33±0.15	0.32±0.1	0.57±0.13	0.44±0.13	0.000
V6(TE)mV	0.52±0.11	0.32±0.09	0.24±0.1	0.29±0.11	0.000
I (ST20)mV	0.23±0.02	0.07±0.04	0.21±0.04	0.29±0.05	0.000
II(ST20)mV	0.66±0.03	0.09±0.05	0.81±0.11	0.87±0.06	0.000
III(ST20)mV	1.01±0.02	0.18±0.07	1.12±0.14	1.35±0.08	0.000
aVR(ST20)mV	1.52±0.02	0.41±0.03	1.48±0.04	1.58±0.04	0.030
aVL(ST20)mV	1.49±0.01	0.66±0.05	1.3±0.09	1.28±0.06	0.000
aVF(ST20)mV	1.2±0.02	0.7±0.06	0.91±0.12	0.96±0.06	0.000
V1(ST20)mV	0±0.04	0±0.09	0±0.08	0±0.06	0.000
V2(ST20)mV	0±0.07	0±0.14	0±0.09	0±0.14	0.000
V3(ST20)mV	0±0.06	0±0.15	0±0.11	0±0.14	0.000
V4(ST20)mV	0±0.05	0±0.13	0±0.09	0±0.12	0.000
V5(ST20)mV	0±0.04	0±0.09	0±0.07	0±0.09	0.000
V6(ST20)mV	0±0.03	0±0.06	0±0.05	0±0.08	0.002
I (ST40)mV	0.01±0.02	0.01±0.04	-0.02±0.05	-0.01±0.05	0.000
II(ST40)mV	0.01±0.03	0.01±0.06	0.06±0.11	0.03±0.06	0.000
III(ST40)mV	0±0.02	0±0.07	0.09±0.14	0.04±0.08	0.000
aVR(ST40)mV	-0.01±0.02	-0.01±0.03	-0.02±0.04	-0.01±0.04	0.088
aVL(ST40)mV	0±0.02	0±0.05	-0.05±0.09	-0.03±0.06	0.000
aVF(ST40)mV	0.01±0.03	0±0.06	0.07±0.12	0.04±0.06	0.000
V1(ST40)mV	0.05±0.04	0.09±0.07	0.04±0.08	0.03±0.06	0.000
V2(ST40)mV	0.12±0.08	0.24±0.15	0.05±0.11	0.06±0.15	0.000
V3(ST40)mV	0.09±0.08	0.2±0.16	0.03±0.12	0.02±0.14	0.000
V4(ST40)mV	0.05±0.06	0.12±0.13	0±0.09	-0.02±0.12	0.000
V5(ST40)mV	0.02±0.04	0.05±0.1	-0.01±0.07	-0.01±0.09	0.000
V6(ST40)mV	0.01±0.03	0.02±0.06	0±0.05	0±0.08	0.015
I (ST60)mV	0.02±0.03	0.01±0.05	-0.02±0.05	0±0.05	0.000
II(ST60)mV	0.02±0.03	0.02±0.06	0.07±0.11	0.04±0.06	0.000
III(ST60)mV	0±0.03	0±0.07	0.09±0.14	0.05±0.08	0.000
aVR(ST60)mV	-0.02±0.03	-0.02±0.04	-0.02±0.05	-0.02±0.04	0.288
aVL(ST60)mV	0.01±0.02	0.01±0.05	-0.05±0.09	-0.02±0.06	0.000
aVF(ST60)mV	0.01±0.03	0.01±0.06	0.08±0.12	0.04±0.06	0.000
V1(ST60)mV	0.06±0.05	0.09±0.08	0.05±0.09	0.04±0.07	0.000
V2(ST60)mV	0.16±0.1	0.26±0.17	0.09±0.13	0.1±0.17	0.000
V3(ST60)mV	0.12±0.1	0.22±0.17	0.06±0.14	0.05±0.16	0.000
V4(ST60)mV	0.07±0.07	0.14±0.15	0.02±0.11	0±0.14	0.000
V5(ST60)mV	0.04±0.05	0.06±0.11	0±0.08	0±0.1	0.000
V6(ST60)mV	0.02±0.04	0.02±0.07	0.01±0.06	0.01±0.08	0.016
I (ST80)mV	0.03±0.03	0.02±0.05	-0.01±0.06	0±0.06	0.000
II(ST80)mV	0.04±0.04	0.03±0.06	0.08±0.12	0.05±0.07	0.000
III(ST80)mV	0±0.03	0.01±0.07	0.09±0.15	0.05±0.08	0.000

aVR(ST80)mV	-0.03±0.03	-0.02±0.04	-0.03±0.05	-0.02±0.05	0.106
aVL(ST80)mV	0.01±0.02	0±0.05	-0.05±0.1	-0.02±0.06	0.000
aVF(ST80)mV	0.02±0.03	0.02±0.06	0.08±0.13	0.05±0.07	0.000
V1(ST80)mV	0.07±0.07	0.1±0.09	0.06±0.1	0.05±0.08	0.000
V2(ST80)mV	0.2±0.13	0.3±0.18	0.15±0.17	0.16±0.2	0.000
V3(ST80)mV	0.17±0.13	0.24±0.19	0.11±0.16	0.09±0.18	0.000
V4(ST80)mV	0.11±0.1	0.15±0.16	0.05±0.13	0.03±0.15	0.000
V5(ST80)mV	0.07±0.07	0.07±0.12	0.02±0.09	0.01±0.11	0.000
V6(ST80)mV	0.04±0.05	0.03±0.08	0.02±0.07	0.02±0.09	0.000

Supplementary Table S6 ECG features among different datasets

	Training set	Validation set	Testing set	External validation set	*P* value
n	445	144	145	86	
Maxmum HR	15.21±0.72	14.26±1.19	12.72±1.06	14.89±1.61	0.545
Minimun HR	25.19±1.19	19.58±1.63	18.65±1.55	19.08±2.06	0.218
Average HR	15.15±0.72	14.91±1.24	14.58±1.21	13.52±1.46	0.415
PR(ms)	152.94±30.13	153.47±27.97	151.82±23.32	147.1±30.64	0.346
P wave(ms)	107.87±25.32	107.95±22.08	106.39±19.12	100.64±24.33	0.069
QRS complex(ms)	104.16±13.28	102.85±10.73	103.56±11.35	104.34±8.57	0.683
T wave(ms)	180.75±28.05	180.23±26.38	181.08±24.65	180.98±32.65	0.994
QT interval(ms)	388.51±38.9	388.47±35.58	389.03±34.26	396.99±38.04	0.276
QTc interval(ms)	432.38±32.95	430.49±30.2	433.01±32.9	433.53±28.89	0.882
QRS axis	40.24±52.89	35.89±51.75	39.89±49.21	26.63±47.66	0.141
RV1+SV5 mv	0.55±0.38	0.52±0.32	0.54±0.31	0.51±0.33	0.615
RV5+SV1 mv	2.06±0.82	2.14±0.87	2.16±0.82	2.05±0.83	0.557
R-PB(ms)	200.38±38.38	199.38±38.69	198.76±34.02	196.44±34.44	0.830
R-P(ms)	139.71±32.08	139.2±33.46	139.5±28.4	142.14±29.66	0.909
R-PE(ms)	93.42±20.39	92.54±22.66	93.54±19.98	95.8±22.95	0.721
R-Q(ms)	49.51±4.45	48.94±3.81	49.54±4.34	49.87±3.52	0.385
R-S(ms)	54.66±10.71	53.91±8.84	54.02±7.94	54.47±6.39	0.817
R-TB(ms)	158.25±29.08	159.3±28.02	158.41±26.32	166.14±26.2	0.120
R-T(ms)	264.53±40.06	266.13±41.54	267.12±36	267.77±42.84	0.848
R-TE(ms)	339±38.66	339.53±35.6	339.5±34.12	347.12±38.73	0.324
Q-P(ms)	93.3±25.46	93.86±26.64	92.33±22.16	94.2±24.41	0.940
S-T(ms)	209.87±40.85	212.22±42.25	213.1±36.96	213.3±42.9	0.775
Q-PB(ms)	154.33±26.28	154.44±24.85	151.86±23.24	148.73±26.12	0.239
S-TE(ms)	284.34±39.48	285.62±36.98	285.48±36.18	292.65±39.76	0.340
I (PB)(mV)	0.06±0.03	0.06±0.03	0.06±0.03	0.06±0.03	0.458
II(PB)mV	0.1±0.04	0.11±0.05	0.1±0.04	0.09±0.04	0.249
III(PB)mV	0.07±0.04	0.07±0.04	0.07±0.04	0.06±0.04	0.188
aVR(PB)mV	0±0	0±0	0±0	0±0	0.650
aVL(PB)mV	0.02±0.02	0.03±0.02	0.03±0.03	0.03±0.03	0.390
aVF(PB)mV	0.08±0.04	0.08±0.04	0.08±0.04	0.07±0.04	0.248
V1(PB)mV	0.04±0.03	0.04±0.03	0.04±0.03	0.03±0.03	0.134
V2(PB)mV	0.05±0.03	0.06±0.03	0.05±0.03	0.05±0.03	0.338
V3(PB)mV	0.06±0.03	0.06±0.03	0.06±0.03	0.06±0.03	0.299
V4(PB)mV	0.06±0.03	0.06±0.03	0.06±0.03	0.06±0.02	0.374
V5(PB)mV	0.06±0.02	0.06±0.03	0.06±0.02	0.06±0.03	0.130
V6(PB)mV	0.05±0.02	0.06±0.03	0.06±0.02	0.06±0.02	0.441
I (PE)mV	0±0	0±0	0±0.01	0±0.01	0.572
II(PE)mV	0±0.01	0±0	0±0.01	0±0	0.489
III(PE)mV	-0.01±0.02	-0.01±0.02	-0.01±0.02	-0.01±0.02	0.307

aVR(PE)mV	-0.07±0.03	-0.08±0.03	-0.08±0.03	-0.07±0.03	0.459
aVL(PE)mV	-0.02±0.02	-0.02±0.02	-0.02±0.02	-0.02±0.02	0.630
aVF(PE)mV	0±0.01	0±0.01	0±0.01	0±0.01	0.980
V1(PE)mV	-0.03±0.03	-0.03±0.03	-0.04±0.03	-0.04±0.03	0.050
V2(PE)mV	-0.01±0.02	-0.01±0.01	-0.01±0.02	-0.01±0.02	0.325
V3(PE)mV	0±0.01	0±0.01	0±0.01	0±0.02	0.457
V4(PE)mV	0±0.01	0±0.01	0±0	0±0.01	0.093
V5(PE)mV	0±0.01	0±0.01	0±0	0±0.01	0.125
V6(PE)mV	0±0.01	0±0	0±0	0±0.01	0.656
I (Q)mV	-0.03±0.05	-0.03±0.03	-0.04±0.11	-0.02±0.03	0.361
II(Q)mV	-0.05±0.08	-0.05±0.08	-0.05±0.07	-0.07±0.11	0.053
III(Q)mV	-0.13±0.24	-0.12±0.21	-0.12±0.23	-0.22±0.29	0.007
aVR(Q)mV	-0.33±0.32	-0.38±0.34	-0.34±0.34	-0.29±0.29	0.200
aVL(Q)mV	-0.05±0.09	-0.04±0.06	-0.04±0.1	-0.03±0.06	0.569
aVF(Q)mV	-0.07±0.13	-0.06±0.12	-0.08±0.13	-0.12±0.18	0.006
V1(Q)mV	-0.16±0.38	-0.16±0.36	-0.12±0.33	-0.23±0.4	0.176
V2(Q)mV	-0.19±0.55	-0.18±0.58	-0.21±0.65	-0.37±0.69	0.057
V3(Q)mV	-0.15±0.46	-0.16±0.53	-0.17±0.54	-0.24±0.51	0.452
V4(Q)mV	-0.07±0.23	-0.07±0.27	-0.06±0.23	-0.08±0.26	0.937
V5(Q)mV	-0.05±0.12	-0.05±0.14	-0.04±0.09	-0.03±0.08	0.557
V6(Q)mV	-0.04±0.06	-0.03±0.05	-0.04±0.06	-0.03±0.05	0.478
I (R)mV	0.57±0.3	0.61±0.3	0.61±0.32	0.56±0.26	0.230
II(R)mV	0.65±0.35	0.68±0.38	0.66±0.36	0.49±0.29	0.001
III(R)mV	0.34±0.31	0.32±0.32	0.33±0.32	0.24±0.24	0.069
aVR(R)mV	0.08±0.09	0.08±0.09	0.08±0.1	0.07±0.07	0.671
aVL(R)mV	0.35±0.28	0.38±0.28	0.38±0.3	0.39±0.24	0.447
aVF(R)mV	0.44±0.33	0.45±0.35	0.43±0.34	0.31±0.26	0.005
V1(R)mV	0.2±0.21	0.22±0.22	0.2±0.22	0.15±0.18	0.078
V2(R)mV	0.58±0.45	0.59±0.46	0.58±0.43	0.54±0.54	0.841
V3(R)mV	0.89±0.64	0.88±0.69	0.92±0.58	0.8±0.74	0.606
V4(R)mV	1.31±0.79	1.31±0.85	1.32±0.7	1.17±0.86	0.486
V5(R)mV	1.29±0.69	1.27±0.72	1.33±0.64	1.29±0.7	0.893
V6(R)mV	1.03±0.53	1.02±0.53	1.1±0.54	1.12±0.56	0.312
I (S)mV	0.13±0.01	0.11±0.01	0.12±0.01	0.1±0.01	0.395
II(S)mV	0.13±0.01	0.15±0.01	0.14±0.01	0.12±0.01	0.028
III(S)mV	0.23±0.01	0.26±0.02	0.26±0.02	0.19±0.02	0.023
aVR(S)mV	0.35±0.02	0.36±0.03	0.37±0.03	0.29±0.03	0.625
aVL(S)mV	0.17±0.01	0.15±0.01	0.17±0.01	0.14±0.01	0.753
aVF(S)mV	0.14±0.01	0.17±0.01	0.16±0.01	0.12±0.01	0.023
V1(S)mV	0.4±0.02	0.43±0.04	0.45±0.04	0.48±0.05	0.080
V2(S)mV	0.73±0.03	0.63±0.05	0.68±0.06	0.73±0.08	0.052
V3(S)mV	0.66±0.03	0.5±0.04	0.62±0.05	0.65±0.07	0.525
V4(S)mV	0.5±0.02	0.39±0.03	0.45±0.04	0.44±0.05	0.117
V5(S)mV	0.32±0.01	0.28±0.02	0.29±0.02	0.27±0.03	0.293

V6(S)mV	0.2±0.01	0.18±0.01	0.19±0.02	0.18±0.02	0.275
I (R')mV	0.02±0	0±0	0±0	0±0	0.719
II(R')mV	0.02±0	0.03±0	0.01±0	0.03±0	0.736
III(R')mV	0.1±0	0.11±0.01	0.1±0.01	0.08±0.01	0.873
aVR(R')mV	0.07±0	0.04±0	0.05±0	0.04±0	0.010
aVL(R')mV	0.03±0	0.01±0	0.02±0	0.01±0	0.089
aVF(R')mV	0.05±0	0.06±0.01	0.08±0.01	0.05±0.01	0.486
V1(R')mV	0.13±0.01	0.05±0	0.09±0.01	0.02±0	0.430
V2(R')mV	0.02±0	0.01±0	0.02±0	0.01±0	0.774
V3(R')mV	0.04±0	0.02±0	0±0	0.09±0.01	0.358
V4(R')mV	0.05±0	0.02±0	0.01±0	0.02±0	0.790
V5(R')mV	0.01±0	0±0	0.02±0	0±0	0.065
V6(R')mV	0.01±0	0±0	0±0	0±0	0.774
I (S')mV	0±0	0±0	0±0	0±0	0.840
II(S')mV	0.01±0	0±0	0±0	0±0	0.642
III(S')mV	0.03±0	0.03±0	0.04±0	0±0	0.406
aVR(S')mV	0±0	0±0	0±0	0±0	0.840
aVL(S')mV	0.01±0	0±0	0±0	0±0	0.685
aVF(S')mV	0.03±0	0.06±0	0.01±0	0±0	0.264
V1(S')mV	0.01±0	0.01±0	0.04±0	0.01±0	0.448
V2(S')mV	0.02±0	0±0	0.16±0.01	0±0	0.150
V3(S')mV	0.01±0	0±0	0±0	0.09±0.01	0.053
V4(S')mV	0.06±0	0±0	0±0	0.06±0.01	0.544
V5(S')mV	0±0	0±0	0±0	0±0	.
V6(S')mV	0±0	0±0	0±0	0±0	0.840
I (TB)mV	0.08±0	0.08±0.01	0.08±0.01	0.08±0.01	0.262
II(TB)mV	0.1±0	0.09±0.01	0.09±0.01	0.08±0.01	0.000
III(TB)mV	0.08±0	0.08±0.01	0.08±0.01	0.08±0.01	0.225
aVR(TB)mV	0.02±0	0.02±0	0.03±0	0.02±0	0.086
aVL(TB)mV	0.07±0	0.08±0.01	0.07±0.01	0.1±0.01	0.041
aVF(TB)mV	0.08±0	0.08±0.01	0.08±0.01	0.08±0.01	0.004
V1(TB)mV	0.09±0	0.1±0.01	0.1±0.01	0.11±0.01	0.849
V2(TB)mV	0.22±0.01	0.25±0.02	0.22±0.02	0.29±0.03	0.711
V3(TB)mV	0.23±0.01	0.25±0.02	0.22±0.02	0.26±0.03	0.856
V4(TB)mV	0.21±0.01	0.22±0.02	0.2±0.02	0.21±0.02	0.139
V5(TB)mV	0.16±0.01	0.16±0.01	0.15±0.01	0.16±0.02	0.042
V6(TB)mV	0.13±0.01	0.12±0.01	0.12±0.01	0.13±0.01	0.030
I (TE)mV	0.08±0	0.08±0.01	0.08±0.01	0.08±0.01	0.262
II(TE)mV	0.1±0	0.09±0.01	0.09±0.01	0.08±0.01	0.000
III(TE)mV	0.08±0	0.08±0.01	0.08±0.01	0.08±0.01	0.225
aVR(TE)mV	0.02±0	0.02±0	0.03±0	0.02±0	0.086
aVL(TE)mV	0.07±0	0.08±0.01	0.07±0.01	0.1±0.01	0.041
aVF(TE)mV	0.08±0	0.08±0.01	0.08±0.01	0.08±0.01	0.004
V1(TE)mV	0.09±0	0.1±0.01	0.1±0.01	0.11±0.01	0.849

V2(TE)mV	0.22±0.01	0.25±0.02	0.22±0.02	0.29±0.03	0.711
V3(TE)mV	0.23±0.01	0.25±0.02	0.22±0.02	0.26±0.03	0.856
V4(TE)mV	0.21±0.01	0.22±0.02	0.2±0.02	0.21±0.02	0.139
V5(TE)mV	0.16±0.01	0.16±0.01	0.15±0.01	0.16±0.02	0.042
V6(TE)mV	0.13±0.01	0.12±0.01	0.12±0.01	0.13±0.01	0.030
I (ST20)mV	0.03±0	0.03±0	0.03±0	0.05±0.01	0.003
II(ST20)mV	0.04±0	0.1±0.01	0.04±0	0.05±0.01	0.183
III(ST20)mV	0.05±0	0.12±0.01	0.05±0	0.08±0.01	0.026
aVR(ST20)mV	0.03±0	0.04±0	0.03±0	0.02±0	0.272
aVL(ST20)mV	0.04±0	0.07±0.01	0.03±0	0.06±0.01	0.006
aVF(ST20)mV	0.05±0	0.11±0.01	0.04±0	0.06±0.01	0.054
V1(ST20)mV	0.05±0	0.08±0.01	0.04±0	0.1±0.01	0.461
V2(ST20)mV	0.11±0.01	0.11±0.01	0.1±0.01	0.12±0.01	0.833
V3(ST20)mV	0.11±0.01	0.13±0.01	0.1±0.01	0.12±0.01	0.954
V4(ST20)mV	0.08±0	0.09±0.01	0.09±0.01	0.11±0.01	0.536
V5(ST20)mV	0.06±0	0.07±0.01	0.06±0	0.07±0.01	0.625
V6(ST20)mV	0.04±0	0.05±0	0.04±0	0.05±0.01	0.394
I (ST40)mV	0.01±0.03	0±0.04	0.01±0.03	-0.01±0.05	0.002
II(ST40)mV	0.02±0.04	0.03±0.09	0.02±0.04	0.02±0.05	0.241
III(ST40)mV	0.01±0.05	0.02±0.11	0.02±0.05	0.03±0.09	0.020
aVR(ST40)mV	-0.01±0.03	-0.01±0.04	-0.01±0.03	-0.01±0.02	0.362
aVL(ST40)mV	0±0.04	-0.01±0.07	-0.01±0.03	-0.02±0.06	0.005
aVF(ST40)mV	0.01±0.05	0.02±0.1	0.02±0.04	0.03±0.07	0.083
V1(ST40)mV	0.06±0.06	0.06±0.08	0.05±0.05	0.06±0.06	0.633
V2(ST40)mV	0.13±0.12	0.13±0.13	0.13±0.11	0.12±0.12	0.945
V3(ST40)mV	0.1±0.12	0.09±0.13	0.1±0.11	0.1±0.13	0.897
V4(ST40)mV	0.05±0.09	0.04±0.1	0.06±0.09	0.05±0.11	0.344
V5(ST40)mV	0.02±0.06	0.01±0.08	0.02±0.06	0.02±0.08	0.528
V6(ST40)mV	0.01±0.04	0±0.05	0.01±0.05	0±0.05	0.561
I (ST60)mV	0.01±0.04	0.01±0.04	0.01±0.03	0±0.05	0.004
II(ST60)mV	0.03±0.05	0.04±0.09	0.03±0.04	0.03±0.06	0.354
III(ST60)mV	0.01±0.05	0.03±0.12	0.02±0.05	0.03±0.1	0.039
aVR(ST60)mV	-0.02±0.03	-0.02±0.04	-0.02±0.03	-0.01±0.03	0.279
aVL(ST60)mV	0±0.04	-0.01±0.07	0±0.03	-0.02±0.07	0.011
aVF(ST60)mV	0.02±0.05	0.03±0.1	0.02±0.04	0.03±0.08	0.160
V1(ST60)mV	0.06±0.07	0.07±0.08	0.06±0.06	0.07±0.06	0.658
V2(ST60)mV	0.16±0.14	0.16±0.15	0.17±0.13	0.16±0.14	0.943
V3(ST60)mV	0.13±0.13	0.12±0.15	0.13±0.12	0.13±0.15	0.775
V4(ST60)mV	0.08±0.1	0.06±0.11	0.08±0.1	0.07±0.12	0.269
V5(ST60)mV	0.04±0.07	0.03±0.09	0.04±0.07	0.04±0.09	0.561
V6(ST60)mV	0.02±0.05	0.01±0.06	0.02±0.06	0.01±0.06	0.594
I (ST80)mV	0.02±0.04	0.02±0.05	0.02±0.04	0.01±0.05	0.008
II(ST80)mV	0.04±0.05	0.05±0.1	0.04±0.05	0.04±0.07	0.366
III(ST80)mV	0.01±0.06	0.03±0.12	0.02±0.05	0.03±0.11	0.083

aVR(ST80)mV	-0.03±0.04	-0.03±0.05	-0.03±0.04	-0.02±0.03	0.166
aVL(ST80)mV	0±0.04	-0.01±0.07	0±0.04	-0.01±0.08	0.029
aVF(ST80)mV	0.03±0.05	0.04±0.11	0.03±0.05	0.03±0.08	0.191
V1(ST80)mV	0.07±0.08	0.08±0.09	0.07±0.07	0.08±0.08	0.735
V2(ST80)mV	0.21±0.16	0.21±0.17	0.22±0.15	0.21±0.16	0.946
V3(ST80)mV	0.17±0.15	0.15±0.16	0.17±0.14	0.17±0.17	0.650
V4(ST80)mV	0.11±0.12	0.09±0.13	0.11±0.11	0.1±0.14	0.174
V5(ST80)mV	0.06±0.08	0.05±0.1	0.06±0.08	0.06±0.1	0.595
V6(ST80)mV	0.03±0.06	0.03±0.06	0.03±0.07	0.03±0.07	0.694

Supplementary Table S7 The Sample Size and the Performance of Model 1 in Patients with Abnormal ECG Phenomenon.

	Sample Size		
Total	82		
Train	53		
Val	16		
Test 1	9		
Test 2	4		
Normal	44		
STEMI	38		
LAD	18		
RCA/LCX	20		
Model 1			
Val+Test 1+ Test 2			29
	Sensitivity	0.895	(34/38)
	Specificity	0.864	(38/44)
	PPV	0.905	(38/42)
	NPV	0.850	(34/40)
	AUC	0.879	(0.797-0.961)
